# Is Vaccine Uptake Related to Health Literacy? A Representative Survey in the Multilingual Region of South Tyrol, Italy

**DOI:** 10.3390/vaccines13060575

**Published:** 2025-05-28

**Authors:** Verena Barbieri, Dietmar Ausserhofer, Stefano Lombardo, Adolf Engl, Giuliano Piccoliori, Timon Gärtner, Christian J. Wiedermann

**Affiliations:** 1 Institute of General Practice and Public Health, Claudiana—College of Health Professions, 39100 Bolzano, Italy; dietmar.ausserhofer@claudiana.bz.it (D.A.); drpiccoliori@gmail.com (G.P.);; 2 Provincial Institute for Statistics of the Autonomous Province of Bolzano—South Tyrol (ASTAT), 39100 Bolzano, Italy; stefano.lombardo@provincia.bz.it (S.L.);; 3 Department of Public Health, Medical Decision Making and Health Technology Assessment, University of Health Sciences, Medical Informatics and Technology, 6060 Hall, Austria

**Keywords:** health literacy, vaccination, multilinguistic, HLS-EU-Q16, Italy, educational level, missing data, mistrust

## Abstract

Background/Objectives: Vaccination rates in South Tyrol, Northern Italy, remain among the lowest in the country. This study investigated whether health literacy is associated with vaccine uptake in this multilingual region. Methods: A representative cross-sectional survey (*n* = 2090) was conducted using the validated European Health Literacy Survey Questionnaire (HLS-EU-Q16) to assess health literacy. Vaccine uptake was evaluated on a 4-point Likert scale and analysed by age group (18–54 years; ≥55 years). Associations were explored using ANCOVA and multivariate logistic regression models. Results: Among younger adults (18–54 years), higher health literacy was significantly associated with greater vaccine uptake, particularly when compared with individuals with missing health literacy data. Health literacy was not a significant predictor for vaccine uptake in the older age group (≥55 years). Instead, vaccine uptake correlated with trust in healthcare providers, the presence of chronic diseases, and educational level. Differences in health literacy were notable across language groups, but these did not interact with vaccination behaviour. Conclusions: Building trust and targeting individuals with lower educational attainment are key strategies for improving vaccination rates across language groups. Although health literacy plays a secondary role, efforts to enhance it, especially among the German-speaking population, are still warranted. Younger individuals with missing health literacy scores, often with a migration background and low trust in healthcare, should be prioritised in vaccination and health literacy campaigns.

## 1. Introduction

Vaccine hesitancy, characterised by a delay in the acceptance or refusal of vaccines despite the availability of vaccination services, is influenced by mistrust in vaccines and health systems, concerns regarding safety, and the spread of misinformation [1,2]. The diverse vaccination policies implemented by various countries, ranging from mandatory to voluntary approaches [2,3], complicate the establishment of a universal strategy to promote vaccination. A comprehensive understanding of the factors influencing vaccine acceptance is crucial to the development of effective communication strategies and interventions aimed at enhancing vaccine uptake in any healthcare system. In Italy, prior to the pandemic, the vaccination of children during their early years was a topic of extensive discussion. The enforcement of compulsory vaccination was a strategy adopted by several countries, including Italy [4,5,6,7,8], to increase immunisation coverage. Although coverage rates have improved, significant regional disparities have persisted. In 2019, childhood vaccination coverage in Italy reached 94% in the second year of life, an increase from the coverage of 2015 [9]. Additionally, the mandatory implementation of hepatitis B vaccination [10] was met with success. For adults, certain vaccinations are recommended and provided for free. Diphtheria, tetanus, and pertussis vaccination is recommended for all adults every ten years. The varicella, measles, rubella, and mumps vaccines and most of the other obligatory childhood vaccinations are recommended for certain risk groups, as well as yearly flu vaccinations. The exact schedule can be found in [11].

South Tyrol, with a total population of approximately 525,000, is an alpine region situated in the northwestern part of Italy, bordering Austria and Switzerland. The region comprises approximately 70 percent German-speaking and 25 percent Italian-speaking inhabitants [12]. These diverse ethno-linguistic groups are served by a unified national healthcare system and service provider. Historically, and even during the pandemic, South Tyrol has been among the regions with the lowest vaccination rates in Italy [13]. In South Tyrol, vaccine hesitancy is shaped by cultural and linguistic identities as well as mistrust in public health authorities [14,15]. There was a notable negative shift in attitudes towards vaccination between 2021 and 2023 [15], necessitating further monitoring and the development of targeted information campaigns.

Empowering citizens for healthy living and monitoring knowledge about health are essential to public health policies. In 2012, the European Health Literacy Survey (HLS-EU) framework was established, including 12 dimensions pertaining to the knowledge, motivation, and competencies for accessing, understanding, appraising, and applying health-related information in the healthcare, disease prevention, and health promotion contexts [16,17]. The HLS-EU was implemented in eight European countries, including Austria and Germany [18,19]. Subgroups characterised by financial deprivation, low social status, limited education, advanced age, frequent doctor visits, and migrant backgrounds [20,21,22,23] exhibited higher proportions of limited health literacy. This suggests a social gradient with variations in health literacy levels across different countries.

While mistrust in public institutions and the spread of misinformation are established predictors of vaccine hesitancy [15], it is important to investigate whether higher health literacy might predict vaccine uptake. The primary objective of our study was to investigate whether health literacy is associated with vaccine uptake in the multilingual context of South Tyrol. Such findings are crucial for planning public health interventions to achieve higher vaccination rates among the German and Italian populations.

The principal research questions of our survey were as follows:

- Is vaccine uptake associated with health literacy?
- Are there differences in vaccine uptake between age groups and linguistic groups?
- Which other sociodemographic factors are associated with vaccine uptake?

## 2. Materials and Methods

### 2.1. Study Design and Sample

Between 1 March and 30 May 2024, a cross-sectional survey was conducted by the Provincial Institute of Statistics (ASTAT; Landesinstitut für Statistik/Istituto Provinciale di Statistica) and the Institute of General Practice and Public Health in the Autonomous Province of Bolzano, South Tyrol. The participants were invited twice by letter to complete the online questionnaire. Participation was voluntary and the participants provided informed consent. All data were anonymised to protect the participants’ identities. An online survey was created using LimeSurvey [24].

The target population was approximately 400,000 individuals aged 18 years and above residing in South Tyrol. Representativity was achieved using a stratified sampling strategy by municipality, citizenship, gender, and age group (18–34, 35–49, 50–64, 65+ years) with the program ‘Surveyselect’ in SAS v9.2. To ensure adequate precision, 3800 individuals were sampled.

### 2.2. Questionnaire

Vaccine uptake was evaluated using a single statement, “I get vaccinated”, with responses recorded on a 4-point Likert scale (1 = “never”, 2 = “seldom”, 3 = “often”, 4 = “always”).

Self-assessed health literacy was measured using the HLS-EU-Q16 questionnaire, which is available in both German [25] and Italian [26,27] versions. This instrument employs Likert-type responses (“very easy”, “fairly easy”, “fairly difficult”, “very difficult”) and includes an “I don’t know” option. For scoring purposes, responses were dichotomised into “easy” (1) and “difficult” (0), with “don’t know/refusal” recoded as missing [25]. The scale score ranged from 0 to 16, with a Cronbach’s alpha of 0.799 for the Italian [26] and of 0.88 for the German [28] versions. Respondents who answered at least 14 items were included, and missing values were assigned a score of 0. Those who answered fewer than 14 items were considered to be missing. The missing response rate was approximately 5% [16]. Three levels of health literacy were defined: inadequate (0–8), problematic (9–12), and adequate (13–16), with missing responses constituting a fourth category.

Sociodemographic variables included age, gender, native language, citizenship, educational level, and employment in the health sector. Residences in the regional capital (Bolzano) were considered urban; all other municipalities were classified as rural, including towns with up to 35,000 inhabitants. Health-related factors include diagnosed chronic diseases and trust in healthcare providers (general practitioners, specialists, and pharmacists). Trust was assessed on an inverse 4-point Likert scale, recoded, and summed to yield a score ranging from 4 (no trust) to 16 (total trust).

The Patient Activation Measure (PAM-10) was utilised per the licence holder in both German and Italian [29,30,31]. The ten questions were answered on a 4-point Likert scale (1 = disagree strongly to 4 = agree strongly) with an additional option, “5 = doesn’t concern me.” The total score was calculated using the Rasch model, with final scores ranging from 0 (no activation) to 100 (total activation). All cases were considered, and the option “5 = doesn’t concern me” was recoded as a mean value according to the PAM-10 manual [32].

### 2.3. Statistical Analysis

Only fully completed questionnaires were included in the statistical analyses. Post-stratification weights were used for all calculations to adjust for non-response bias and ensure that the sample was representative of the target population. Descriptive statistics were used to describe the measured variables. For continuous variables, the mean ± standard deviation (SD) was computed; for categorical variables, absolute and relative numbers were reported. Group comparisons were performed using chi-square tests for nominal and ordinal data and Mann–Whitney and Kruskal–Wallis tests for metric data. Associations between the variables were assessed using Kendall’s tau-b test.

Data were split into two age groups—18–54 years and 55 years or older—and analysed separately.

To explore the association between dichotomised vaccine uptake (1 = often/always; 0 = seldom/never) and sociodemographic variables, categorised health literacy (adequate, problematic, inadequate, missing), and health-related factors, a stepwise multivariate binary logistic regression model was used with a threshold probability for inclusion of 0.05 and for exclusion of 0.1. A minimal sample size of 898 was needed for an assumed probability of 0.6, odds ratio of 1.25, type one error of 5%, and power of 0.9.

The associations between the HLS-EU-Q16 score and vaccine uptake, corrected for other predictors, were analysed using a univariate ANCOVA model with covariates, assuming that the ANCOVA was robust even for non-normally distributed data. A minimal sample size of *n* = 459 was required for a medium effect size, error probability of 0.05, power of 0.95, 4 × 4 groups, and three covariates.

Significance was considered as follows for *p*-values: *p* < 0.05 (*) slightly significant, *p* < 0.01 (**) medium significant, and *p* < 0.001 (***) highly significant. Post hoc tests were performed using Bonferroni correction. All analyses were conducted using SPSS version 27.0, and the power calculations were performed using G*Power 3.1.9.4.

## 3. Results

In total, 2090 adults from the general population participated in the survey, resulting in a response rate of 55%. All participants responded to the question regarding vaccine uptake: 162 (8.0%) reported never vaccinating, 662 (31.7%) reported seldom vaccinating, 570 (27.3%) reported often vaccinating, and 690 (33.0%) reported being vaccinated.

The reliability of the two questionnaires, HLS-EU-Q16 (Cronbach’s alpha = 0.89, missing rate = 21.1%) and PAM-10 (Cronbach’s alpha = 0.81, with missing values considered 2.5), was confirmed.

Among the participants, 50.9% were identified as female, 20.3% resided in urban areas (regional capital), 90.2% held Italian citizenship, 10.3% were employed in the health sector, and 35.7% had a chronic disease. Educational attainment varied, with 21.1% having, at most, a middle school degree, 31.3% having a vocational school degree, 26.1% having a high school degree, and 20.9% having a university degree. Regarding the language, 63.6% reported German as their mother tongue, 22.9% Italian, 3.8% Ladin, and 9.7% indicated having more than one or another mother tongue.

### 3.1. Vaccine Uptake and Health Literacy by Age Groups

**Figure 1:**
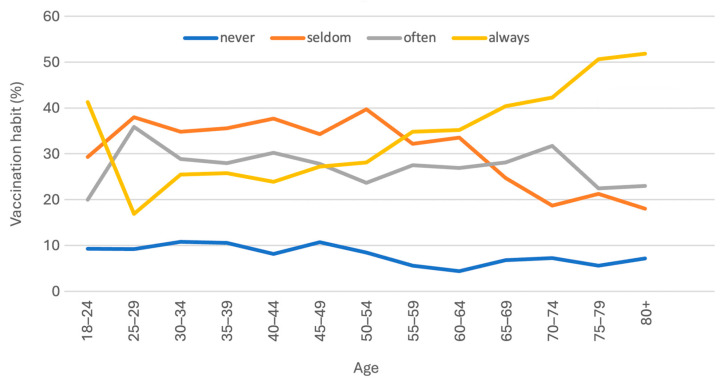
Vaccine uptake for 5-year age groups (total population n = 2090).

**Figure 2:**
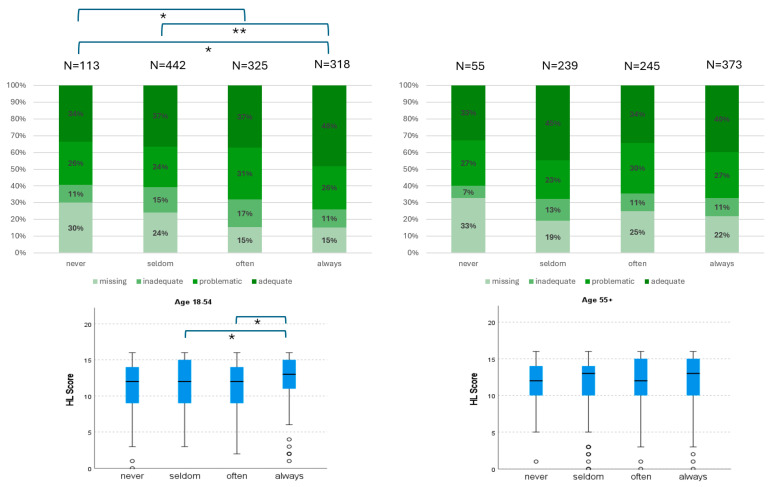
Health literacy distribution and vaccine uptake by age group. Stacked bar charts (**left**) show the distribution of HLS-EU-Q16 categories—missing, inadequate, problematic, and adequate—across vaccine uptake groups (“never”, “seldom”, “often”, “always”) in the younger ((**top**): 18–54 years; *n* = 113/442/325/318) and older ((**bottom**): 55+ years; *n* = 55/239/245/373) age groups. Boxplots (**right**) display the corresponding HLS-EU-Q16 scores (range: 0–16) for each group. Significant differences in health literacy categories and scores were observed across vaccine uptake groups in the younger age group (*p*  <  0.05 * to *p*  <  0.01 **, Bonferroni corrected) but not in the older age group (n.s. = not significant).

Figure 1 illustrates the association between vaccine uptake and age. Among participants aged 55 years and older, there was an increase in the number of individuals who reported “always” receiving vaccinations, while the number of those who reported “seldom” receiving vaccinations decreased. A statistically significant difference was observed between the two age groups, 18–54 years (n = 1178) and 55+ years (n = 912), in terms of vaccine uptake (*p* < 0.001) and HLS-EU-Q16 score (*p* = 0.005), although no significant difference was found in the HLS-EU-Q16 categories (Figure 2). In the younger cohort, 9.6% reported “never” vaccinating, 35.9% “seldom”, 27.6% “often”, and 27.0% “always.” Conversely, in the older cohort, 6.0% reported “never” vaccinating, 26.2% “seldom”, 26.9% “often”, and 40.8% “always.”

The mean and standard deviation of the overall HLS-EU-Q16 score was 11.9 ± 3.41 (n = 1649). In the younger age group, the HLS-EU-Q16 score mean + -SD was 11.8 ± 3.42, whereas in the older age group, it was 12.0 ± 3.39. Of the 2090 patients, 442 (21.1%) were not evaluated for the HLS-EU-Q16 score, 266 (12.7%) had an inadequate HLS-EU-Q16 score, 559 (26.7%) had a problematic score, and 824 (39.4%) had an adequate score. In the younger age group, 19.8% were missing, 14.0% had inadequate scores, 26.6% had problematic scores, and 39.6% had adequate scores. In the older age group, 22.7% were missing, 11.1% had inadequate scores, 27.0% had problematic scores, and 39.3% had adequate scores.

Figure 2 illustrates the distribution of the HLS-EU-Q16 (*n* = 2090) and HLS-EU-Q16 scores (*n* = 1649) across vaccination categories for both age cohorts. In the younger age cohort, differences in the HLS-EU-Q16 categories between vaccination groups were highly significant (*p* < 0.001), whereas no significant differences were observed in the older cohort. For the younger cohort, adequate HLS-EU-Q16 increased with higher vaccine uptake, whereas the absence of HLS-EU-Q16 information decreased with higher vaccine uptake. Post hoc analyses with Bonferroni correction identified significant differences between the groups “never” vs. “often” (*p_corrected_* = 0.036), “seldom” vs. “always” (*p_corrected_* = 0.006), and “never” vs. “always” (*p_corrected_* = 0.018). In contrast, no significant differences in the HLS-EU-Q16 categories concerning vaccine uptake were observed in the older cohort.

Regarding the HLS-EU-Q16 score, the younger cohort exhibited a significant difference in relation to vaccine uptake (*p* = 0.004), with post hoc tests revealing differences between “often” vs. “always” (*p_corrected_* = 0.004) and “seldom” vs. “always” (*p_corrected_* = 0.027). No such differences were observed in the older cohort.

### 3.2. Dichotomised Vaccine Uptake and Possible Predictors in Both Age Groups

The associations between dichotomised vaccine uptake and potential predictors for both age cohorts are presented in Table 1. Among the metric variables, trust in healthcare personnel exhibited a strong positive correlation with increased vaccine uptake in both age groups, whereas PAM-10 score did not demonstrate a significant association with vaccine uptake. Notably, in the older age group, advanced age strongly correlated with higher vaccine uptake.

Regarding categorical variables, only the presence of a chronic disease and educational level were significantly associated with vaccine uptake in the older age group. Post hoc analyses with Bonferroni correction identified significant differences for the vocational school/high school pair (*p_corrected_* = 0.012).

In the younger age group, there was a slight positive association of vaccine uptake with female gender, urban residency, and the presence of a chronic disease. Additionally, a positive association was observed with Italian nationality, employment in the health sector, and the presence of a chronic disease. Furthermore, vaccine uptake was highly associated with educational level and mother tongue.

For educational level, post hoc tests with Bonferroni correction revealed significant differences in the following pairs: middle school or less/university or more (*p_corrected_* = 0.018), vocational school/high school (*p_corrected_* < 0.001), and vocational school/university or more (*p_corrected_* < 0.001). For the mother tongue, post hoc tests with Bonferroni correction identified significant differences in the following pairs: German/Italian (*p_corrected_* = 0.018), German/Ladin (*p_corrected_* = 0.048), Italian/Ladin (*p_corrected_* < 0.001), and Italian/other (*p_corrected_* < 0.001).

### 3.3. Participants with Missing HLS-EU-Q16 Score

In this study, 21.1% of cases exhibited “missing” HLS-EU-Q16 scores, a proportion notably higher than those reported in other studies conducted in Germany [27] and Italy [26]. Upon examining this specific cohort, significant differences were observed in the younger age group when compared to 78.9% of the participants with available HLS-EU-Q16 scores. These differences in missing HLS-EU-Q16 scores were evident in terms of educational attainment (middle school: 27.1%; vocational school: 24.4%, high school: 19.8%, university: 12.1%; *p* < 0.001), nationality (Italian, 18.2%; others, 31.7%; *p* < 0.001), mother tongue (German 20.2%, Italian 14.0%, Ladin 9.8%, others 29.3%; *p* = 0.001), and employment in the health sector (9.5%, no 21.4%; *p* = 0.001). In the older age group, significant differences in missing HLS-EU-Q16 scores were found in educational level (middle school, 31.7%; vocational school, 20.9%; high school, 19.3%; university, 6.8%; *p* < 0.001) and mother tongue (German, 25.3%; Italian, 17.3%; Ladin, 14.8%; others, 27.0%; *p* = 0.047). No significant differences were observed in gender, residency, or chronic disease status in either age group. Additionally, nationality and employment in the health sector did not significantly affect the incidence of missing HLS-EU-Q16 scores in the older age groups.

In examining the metric variables, it was observed that within the younger age cohort, there was no significant difference in age between participants with and without missing HLS-EU-Q16 scores. However, the PAM-10 score was notably lower in the group with missing data (mean ± SD, 53.1 ± 11.5 vs. 58.0 ± 12.4; *p* < 0.001), as was the trust score (12.2 ± 2.4 vs. 12.7 ± 2.1; *p* = 0.001). Conversely, in the older age cohort, participants with missing HLS-EU-Q16 scores were significantly older (70.8 ± 10.4 vs. 66.8 ± 9.3; *p* < 0.001) and exhibited a significantly lower trust score (12.5 ± 2.2 vs. 12.9 ± 2.0; *p* = 0.023), while the PAM-10 score did not differ between the groups.

### 3.4. Logistic Regression Model to Explain Dichotomised Vaccine Uptake

A logistic regression model was conducted to examine the association between dichotomised vaccine uptake (0 = “never/seldom”; 1 = “often/always”) and categorised health literacy (HLS-EU-Q16: adequate, problematic, inadequate, missing) in both age groups. Further predictors are listed and tested for association with the dependent variable in Table 1.

In the younger group, urban residence was strongly correlated with the German (ρ = −0.384, *p* < 0.001) and Italian mother tongues (ρ = 0.351, *p* < 0.001). Similarly, Italian citizenship was correlated with the German mother tongue (ρ = 0.357, *p* < 0.001) and other/non-Italian mother tongues (ρ = −0.705, *p* < 0.001). To avoid multicollinearity, urban residence and citizenship were excluded from the regression model. All other predictor variables showed low correlations (ρ < 0.3) or were uncorrelated. In the older age group, no strong correlations were observed among the predictors.

Table 2 presents the results of the logistic regression in terms of odds ratios (OR). In the younger age group (18–54 years), the logistic regression model detected a positive effect of working in the health sector (OR = 1.53 [1.03;2.27]), having a chronic disease (1.38 [1.02;1.87]), and trust in health care personnel (OR = 1.28 [1.20;1.36]). The educational levels “middle school” (OR = 0.62 [0.39;0.98]) and “vocational school” (OR = 0.46 [0.33;0.64]) had a negative effect, while “high school” had no effect when university was used as a baseline. Italian mother tongue had a positive effect (OR = 1.43 [1.01;2.01]), and the Ladin mother tongue had a negative effect (OR = 0.41 [0.22;0.76]) when German mother tongue was used as baseline. Other/more than one mother tongue was not significant. While a problematic or inadequate HLS-EU-Q16 score had no effect, a missing HLS-EU-Q16 score had a significantly negative effect (OR = 0.69 [0.49;0.97]) on vaccine uptake when an adequate HLS-EU-Q16 score was used as the baseline.

In the older age group (55+ years), vaccine uptake increased with age (OR = 1.02 [1.00;1.04]), trust in healthcare personnel (OR = 1.20 [1.12;1.29]), and chronic diseases (OR = 1.58 [1.17;2.14]), while it was negatively affected by vocational education level (OR = 0.55 [0.34;0.89]). The educational levels of middle and high school were not significant.

### 3.5. ANCOVA Analysis of Health Literacy Scores and Their Association with Vaccine Uptake

To further explore the relationship between health literacy and vaccine uptake, we analysed the continuous HLS-EU-Q16 score, acknowledging a missing data rate of 21.1%. The mean ± SD of the HLS-EU-Q16 score across the four vaccination uptake categories was as follows: “never” 11.70 ± 3.52, “seldom” 11.71 ± 3.57, “often” 11.62 ± 3.37, and “always” 12.32 ± 3.23, with a statistically significant overall difference (*p* = 0.004). Significant variations in HLS-EU-Q16 scores were also observed across mother tongue groups (*p* < 0.001), with mean scores as follows: German 11.50 ± 3.49, Italian 12.55 ± 3.23, Ladin 12.33 ± 2.60, and other/more than one language 12.64 ± 3.13. No statistically significant differences in HLS-EU-Q16 scores were found with respect to gender, residency, nationality, educational level, employment in the health sector, or the presence of a chronic disease.

Correlation analyses revealed a slight positive association between the HLS-EU-Q16 score and age (Spearman’s ρ = 0.086, *p* < 0.05), and moderate positive correlations with the PAM-10 score (ρ = 0.338, *p* < 0.001) and trust in healthcare personnel (ρ = 0.255, *p* < 0.001).

To examine differences in HLS-EU-Q16 scores across vaccination uptake categories, a univariate analysis of covariance (ANCOVA) was conducted. Although the HLS-EU-Q16 score was not normally distributed, the ANCOVA approach was considered robust to such deviations, thus supporting the appropriateness of the method.

The univariate analysis of covariance revealed no significant age differences between the vaccination groups. However, significant differences were observed in the PAM-10 scores and trust in healthcare personnel across groups, rendering them unsuitable as covariates. Table 3 provides a detailed overview of the modelling approach.

ANCOVA was employed with the HLS-EU-Q16 score (*n* = 1649) as the dependent variable and the four vaccine uptake categories as fixed factors. Initially, no covariates were included in the analysis. A significant difference was identified between the vaccine uptake groups (Table 3, uncorrected *p*-values). Post hoc tests indicated significant differences between the “seldom” and “always” groups (*p* = 0.029) and the “often” and “always” groups (*p* = 0.009). Age was subsequently included as a covariate. The assumption of homogeneity of the regression slopes was met because the interaction term was not statistically significant. The effect of age was minor and only marginally significant, with post hoc tests revealing differences between the “seldom” and “always” groups (*p* = 0.021) and the “often” and “always” groups (*p* = 0.007).

In the third step, mother tongues were introduced as independent random factors, with German as the baseline. No significant interaction was observed between vaccine uptake and mother tongue with respect to the HLS-EU-Q16 score, although an interaction was identified between age and mother tongue. Consequently, age was excluded from the model. The “never” category was no longer significant. Post hoc tests confirmed significant differences between the “often” and “always” groups (*p* = 0.038). All mother tongues exhibited a positive effect compared to the German mother tongue. Post hoc tests with Bonferroni correction confirmed the positive effect of Italian (*p* < 0.001) and “other/more than one” mother tongue (*p* = 0.009), but not of the Ladin mother tongue.

**Figure 3:**
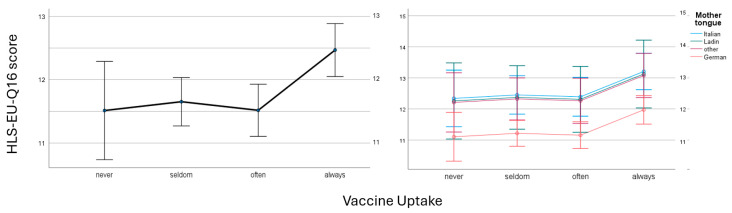
Estimated marginal means of HLS-EU-Q16 scores by vaccine-uptake group. (**Left**) ANOVA model without covariates. (**Right**) ANCOVA model including the mother tongue as a random factor. German mother tongue served as a reference. No interaction was found between vaccine uptake and the mother tongue.

Table 3 presents the three models: the first without covariates, the second including age, and the third including mother tongue. The estimated marginal means are depicted in Figure 3, which illustrates the higher HLS-EU-Q16 score in the “always” vaccinating group and the lower HLS-EU-Q16 score for the German mother tongue compared to other mother tongues. Figure 3 also demonstrates the absence of an interaction between the mother tongue and vaccine uptake in the HLS-EU-Q16 score.

## 4. Discussion

This study examined the association between health literacy and vaccine uptake in a representative sample of the adult population in South Tyrol, a multilingual region in Northern Italy. The findings suggest that health literacy is linked to vaccine uptake among younger adults (18–54 years) but not among those aged 55 years and older. In the younger cohort, higher HLS-EU-Q16 scores were significantly correlated with more frequent vaccination behaviour, particularly when compared to individuals lacking health literacy data. Conversely, vaccine uptake in the older cohort was primarily associated with age, trust in healthcare providers, educational level, and the presence of chronic disease. Trust in healthcare professionals emerged as a robust and consistent predictor of vaccine uptake across both age groups. Additionally, linguistic differences were observed in the HLS-EU-Q16 scores, with individuals reporting Italian or other/non-German mother tongues exhibiting higher health literacy than German-speaking respondents. However, these language-related differences did not result in significant interactions with vaccination behaviour. Notably, a substantial proportion of younger individuals lacked evaluable health literacy data, and this subgroup was characterised by lower trust in healthcare and lower patient activation (PAM-10), indicating a potentially vulnerable group that may benefit from targeted interventions.

The primary aim of this study was to explore whether health literacy is associated with vaccination uptake. The results were not as conclusive as anticipated and thus warrant cautious interpretation. Sørensen et al. [15] identified limited evidence regarding the relationship between health literacy and vaccine uptake. Their meta-analysis revealed positive, negative, and non-significant associations. Elderly individuals were more likely to receive vaccinations when they had higher health literacy, whereas European individuals exhibited greater vaccine hesitancy when they had higher health literacy. Parental health literacy did not correlate with increased vaccine uptake among children. Overall, there is a paucity of representative studies examining the associations between standardised instruments for measuring health literacy and vaccine uptake. The HLS-EU-Q16 can serve as a tool for European countries.

### 4.1. Vaccine Uptake and Health Literacy by Age Group

Preliminary observations revealed a peak in vaccination behaviour at 54 years of age. A detailed analysis of the data revealed significant differences between the two age groups across nearly all variables, except for health literacy. The first notable finding was that higher vaccine uptake was correlated with greater health literacy in the younger age group, but this correlation was not evident in the older age group. Consequently, irrespective of other factors, it cannot be asserted that improved health literacy in individuals aged 55 years or older is linked to increased vaccine uptake. Results from logistic regression modelling indicated that higher vaccine uptake in this age group was associated with having a chronic disease, trust in healthcare personnel, and not possessing a vocational education level. From the first two predictors, it can be inferred that older individuals generally receive more assistance from healthcare personnel; thus, higher vaccine uptake is attributable to a robust healthcare supply. In the context of vaccination, the health literacy of older individuals may be of limited significance. This finding may mediate the assertion of previous studies [21,22,23], where older individuals were less likely to possess adequate health literacy, and lower health literacy was influenced by frequent doctor visits and low functional literacy.

A second key observation was the 21.1% rate of missing HLS-EU-Q16 scores, whereas most studies [21,26,27,33] report a rate of less than 5%. Our objective was to investigate the implications of this “missing” subgroup on vaccine uptake. In the younger demographic group, the absence of responses was a significant predictor of low vaccine uptake in the logistic regression model. This subgroup within the younger population was characterised by lower educational attainment, a higher likelihood of non-Italian citizenship, more frequent use of a non-Italian language, predominantly non-employment in the health sector, a lower PAM-10 score, and increased mistrust among healthcare personnel. Given that studies [20,21,22,23] have associated these traits with individuals possessing low or inadequate HLS-EU-Q16 scores, it is plausible that the “missing” group also lacks health literacy. Consequently, this group should be considered a target for strategic health literacy and vaccination initiatives.

This subgroup presents another noteworthy feature of the dataset. By incorporating “missing” as a fourth category in the HLS-EU-Q16 score, we were able to conduct a comprehensive analysis of the entire dataset. Our findings revealed a significant difference between the two age groups concerning the categorised HLS-EU-Q16 score, with the younger group demonstrating higher health literacy rates and the older group exhibiting higher rates of missing data. Previous studies [19,20,21,22,23] have reported that health literacy is generally lower in older individuals than in younger individuals. However, our analysis of the HLS-EU-Q16 score alone did not corroborate this finding, as no association with age was observed in the overall dataset. If the “missing” category in our categorical analysis represents the group with the lowest health literacy, we are able to confirm the result.

### 4.2. Linguistic Differences in Health Literacy and Vaccine Uptake

Another important finding relates to the role of health literacy and native language in the context of vaccination. Tiller et al. [28] employed the HLS-EU-Q16 to evaluate health literacy levels within the German population, revealing that 55.8% of individuals possessed adequate health literacy, 31.9% exhibited problematic health literacy, and 12.3% demonstrated inadequate health literacy. Variations in health literacy were linked to educational attainment and physical activity, but not to gender or age. Among older adults [34], women generally achieved higher HLS-EU-Q16 scores, and disparities were observed among individuals with specific chronic conditions. In Italy, a representative survey utilising the HLS-EU was conducted in 2016 [26] and the Italian version of the HLS-EU-Q16 was validated in 2019 [27]. The instrument was deemed reliable, although the results differed from those of other published studies. Specifically, health literacy levels measured with HLS-EU tools in the Italian population were generally lower than those reported in other population-based studies [33,35]. Notably, when alternative health literacy assessment instruments were employed, the opposite trend emerged, with Italy exhibiting comparatively higher health literacy levels [19].

In Germany [27], adequate health literacy rates were approximately 55.8%, whereas in Italy [26] they were approximately 33%. The authors attributed these differences to cultural factors. In our dataset, we observed an opposite effect. When considering only the 78.9% of participants with an available HLS score, the corresponding percentages of an adequate HLS-EU-Q16 score were 43.9% in the German group, 60.5% in the Italian group, 47.8% in the Ladin group, and 64.6% in the more than one/other language group. Including 21.1% of missing values, the corresponding percentages were 34.1%, 50.9%, 42.3%, and 46.0%. It is challenging to determine which percentages should be compared with the German [26] and Italian [27] results. If the “missing” group is considered a group with inadequate health literacy, the difference between our results and those from Germany is approximately −20%, and the difference between our Italian group and results from Italy is about 18%. Nonetheless, our findings are contrary to national studies, as in our study, the health literacy of Germans is significantly lower than that of Italians. A plausible explanation for this result may be the unique linguistic situation in South Tyrol. The German minority in Italy requires targeted health literacy information, as it is typically not informed by national-level health literacy information. Conversely, the health literacy level in the Italian South Tyrolean population was considerably higher than that reported in [27]. This intriguing result warrants further investigation.

In previous studies, we identified that younger individuals in South Tyrol tended to exhibit greater vaccine hesitancy [13,14,15]. We emphasise the necessity of culturally and linguistically tailored communication strategies to effectively address and mitigate this hesitancy. The current study corroborates minor linguistic variations in vaccine uptake yet reveals significant linguistic disparities in health literacy. These disparities persist across vaccination groups, indicating that the interaction between health literacy and vaccination is not evident in a linguistic context. Consequently, enhancing health literacy and vaccine uptake should be approached as distinct issues. While a specialised strategy to improve health literacy is generally required for the German-speaking population, fostering trust in vaccination is a challenge that must be addressed across all linguistic groups, with particular attention to the Ladin-speaking community.

### 4.3. Implications for Public Health Communication and Interventions

Results raise the question of whether vaccine uptake can be effectively enhanced by implementing targeted programmes for individuals with lower educational attainment. Interventional studies are needed. Additionally, fostering trust in health care professionals should be a priority across all age groups. These findings align with those of studies that identified the establishment of trust in healthcare institutions and the provision of accessible and transparent information as crucial factors in promoting public confidence and adherence to vaccination initiatives [14,15].

### 4.4. Comparison with Previous Research

Comparing our findings with recent research conducted in Italy, both congruencies and discrepancies emerged. The focus was on health literacy and vaccination in Italy, particularly through the creation of a tool to evaluate vaccine literacy (VL) in 2020 [35]. In [36], authors illustrated that perceptions of prospective COVID-19 vaccines, along with vaccination beliefs, were largely positive and significantly correlated with functional and interactive-critical VL scales. The influence of health literacy on parental decisions regarding childhood vaccination was examined in [37]. No correlation was found between health literacy levels and an increased inclination towards preventive strategies, likely due to the multifaceted nature of the issue, as health literacy alone does not provide reassurance for the numerous enquiries posed by families. In [38], many participants, especially those with lower educational levels and migration backgrounds, reported challenges in processing vaccination information. In [39], the vaccine confidence index (VCI) did not show a significant association with health literacy and knowledge of vaccine-preventable diseases. Conversely, studies [40,41] indicate that higher health literacy is positively associated with increased vaccine hesitancy.

In Germany [42], particular attention was given to vaccine literacy by proposing strategies for effectively managing perceptions and misperceptions across various communication levels and how the internet can be utilised to achieve this objective. A compilation of internet resources for patients and healthcare personnel is recommended. Individuals who were undecided [43] and those more inclined to vaccinate were generally less likely to report a satisfactory subjective level of information and competence in evaluating COVID-19 vaccination information and decision-making. Unvaccinated individuals without vaccination intentions are more likely to report information perceived as untrustworthy or incorrect. Individuals with lower educational levels, younger individuals, and those with migrant backgrounds reported lower levels of health literacy.

Although the impact of health literacy on vaccine uptake in our sample was not as pronounced as initially expected, it may be beneficial to promote health literacy within the general population to foster trust in health care providers. Regardless of other factors, building trust remains the most crucial and effective tool for enhancing vaccine uptake. Further research on the relationship between trust and health literacy is required.

### 4.5. Strengths and Limitations

This study had several strengths. It was grounded in a large, representative sample of the adult population in South Tyrol, achieved through stratified random sampling. The use of a validated instrument for health literacy (HLS-EU-Q16) facilitates comparability with other European studies. Additionally, the inclusion of both categorised and continuous HLS-EU-Q16 scores, along with the analysis of missing data as the fourth category, offers novel insights into vulnerable subgroups.

Nevertheless, this study has several limitations that must be acknowledged. The cross-sectional design precludes causal inference between health literacy and vaccine uptake. The relatively high proportion of missing HLS-EU-Q16 responses (21.1%) may have introduced selection bias, although efforts were made to analyse and characterise this subgroup. Self-reported vaccine uptake, assessed using a single Likert-scale item, may be subject to social desirability and recall bias. Furthermore, although the sample was linguistically and geographically representative, the findings may not be generalisable to other regions of Italy or countries with different healthcare systems or cultural dynamics.

Finally, the question: “I get vaccinated” is very general and not specifically pointing to a specific vaccine. Our aim was to understand the vaccine uptake of adult South Tyrolians, since a list of recommended vaccines is available and the vaccines are provided for free. Capturing a general picture of vaccination attitudes cannot substitute for specific investigations on the uptake of individual vaccines.

## 5. Conclusions

Despite ongoing efforts, low vaccination rates remain a pressing public health concern in South Tyrol. While trust in healthcare providers and targeted information campaigns for individuals with lower educational attainment appear to be critical across all linguistic groups, the role of health literacy in vaccine uptake was less pronounced than anticipated. Nevertheless, improving health literacy, particularly among the German-speaking population, remains a relevant goal from a broader public health perspective.

One particularly vulnerable subgroup identified in this study was comprised of younger individuals with missing HLS-EU-Q16 scores. This group, characterised by lower educational levels, higher mistrust in healthcare, and often a background of migration, demonstrated significantly lower vaccine uptake. Tailored vaccination and health literacy initiatives should prioritise this subgroup to address the existing disparities and enhance immunisation coverage.

## Figures and Tables

**Table 1:** Demographic and other characteristics of dichotomised vaccine uptake per age group.

Variable	Total *n* = 2090 (100%)	Vaccine Uptake					
		Age 18–54 (*n* = 1178)			Age 55+ (*n* = 912)		
		Never/Seldom *n* = 535 (45.4%)	Often/Always *n =* 643 (54.6%)		Never/Seldom *n* = 294 (32.2%)	Often/Always *n =* 618 (67.8%)	
	Mean (SD)	Mean (SD)		*p*-value ^1^	Mean (SD)		*p*-value ^1^
Age	50.9 (17.90)	38.4 (10.22)	37.5 (10.56)	n.s.	66.2 (9.34)	68.4 (9.85)	<0.001
PAM-10 score	56.4 (13.01)	56.5 (13.58)	57.5 (12.82)	n.s.	54.8 (12.64)	56.0 (12.78)	n.s.
Trust in health care providers	12.7 (2.13)	12.0 (2.29)	13.1 (1.94)	<0.001	12.2 (2.15)	13.0 (1.97)	<0.001
		%		*p*-value ^2^	%		*p*-value ^2^
Gender				0.036			n.s.
male		48.5	51.5		34.4	65.6	
female		42.4	57.8		30.2	69.8	
Residence				0.019			n.s.
urban		38.2	61.8		29.1	70.9	
rural		47.1	52.9		33.2	66.8	
Education				<0.001			<0.001
middle school or less		50.4	49.6		31.4	68.6	
vocational school		58.1	41.9		38.7	61.3	
high school		41.3	58.7		24.6	75.4	
university or more		34.0	66.0		29.1	70.9	
Nationality				0.003			n.s.
Italian		43.9	56.1		32.0	68.0	
other		57.0	43.0		34.9	65.1	
Mother tongue				<0.001			n.s.
German		45.5	54.5		33.0	72.2	
Italian		34.1	65.9		27.8	72.2	
Ladin		64.7	35.3		39.3	60.7	
other		53.9	46.1		47.2	52.8	
Work in the health sector				0.002			n.s.
yes		33.3	66.7		34.3	65.7	
no		47.1	52.9		32.0	68.0	
Chronic diseases				0.046			<0.001
yes		39.8	60.2		26.6	73.4	
no		46.9	53.1		39.0	61.0	

^1^ Mann–Whitney-test; ^2^ Chi-Square-test; n.s., not significant.

**Table 2:** Logistic regression models were used to explain the higher vaccine uptake in the age groups 18–54 years (*n* = 1078) and 55+ years (*n* = 912).

Predictor	Age Group 18–54 Years Nagelkerkes’ R^2^ = 0.158		Age Group 55+ Years Nagelkerkes’ R^2^ = 0.115	
	OR [95%CI]	*p*-Value	OR [95%CI]	*p*-Value
Constant	0.09	<0.001	0.06	<0.001
Age			1.02 [1.00;1.04]	0.024
Female gender		n.s.		
Working in the health sector	1.53 [1.03;2.27]	0.036		
Chronic disease	1.38 [1.02;1.87]	0.035	1.58 [1.17;2.14]	0.003
Middle school or less ^1^	0.62 [0.39;0.98]	0.041		n.s.
Vocational school ^1^	0.46 [0.33;0.64]	<0.001	0.55 [0.34;0.89]	0.015
High school ^1^		n.s.		n.s.
Trust in health care personal	1.28 [1.20;1.36]	<0.001	1.20 [1.12;1.29]	<0.001
Italian ^2^	1.43 [1.01;2.01]	0.042		
Ladin ^2^	0.41 [0.22;0.76]	0.005		
Other/more than one ^2^		n.s.		
HLS-EU-Q16 missing ^3^	0.69 [0.49;0.97]	0.034		
HLS-EU-Q16 inadequate		n.s.		
HLS-EU-Q16 problematic		n.s.		

^1^ Educational level: baseline university; ^2^ mother tongue: baseline German; ^3^ HLS-EU-Q16: baseline adequate. n.s.=not significant

**Table 3:** Coefficients from ANCOVA models with HLS-EU-Q16 score as the dependent variable and vaccine uptake as the fixed factor.

Independent Variables	No Covariates		Covariate: Age		Random Factor: Mother Tongues	
	Beta (95% CI)	*p*-Value	Beta (95% CI)	*p*-Value	Beta (95% CI)	*p*-Value
Vaccination						
never ^1^	−0.95 [−1.84;−0.07]	0.034	−0.96 [−1.84;−0.85]	0.032		n.s.
seldom ^1^	−0.82 [−1.38;−0.25]	0.005	−0.84 [−1.41;0.28]	0.003	−0.75 [−1.32;−0.19]	0.009
often ^1^	−0.95 [−1.54;−0.36]	0.002	−0.97 [−1.55;−0.38]	0.001	−0.81 [−1.40;−0.23]	0.006
Age			0.024 [0.002;0.045]	0.033		
Mother tongue						
Italian ^2^					1.234 [0.651;1.817]	<0.001
Ladin ^2^					1.15 [0.12;2.19]	0.029
Other/more than one ^2^					1.10 [0.43;1.78]	0.001

^1^ Vaccination: baseline always; ^2^ mother tongue: baseline German.

## Data Availability

Data are available from the corresponding author upon request.

## Abbreviations

The following abbreviations are used in this manuscript:

HLS-EU-Q16	European Health Literacy Survey (16 Questions)
VL	Vaccine literacy
VLI	Vaccine literacy index
PAM-10	Patient activation measure
ASTAT	Landesinstitut für Statistik/Istituto Provinciale di Statistica
SD	Standard deviation
